# Supplementary material for: White matter connectivity in brain networks supporting social and affective processing predicts real-world social network characteristics
Source: Commun Biol. 2022 Oct 3;5:1048. doi: 10.1038/s42003-022-03655-8 (PMC9529948; doi:10.1038/s42003-022-03655-8)
Supplement: Supplementary file 2 — Supplementary Information [file 42003_2022_3655_MOESM2_ESM.pdf]

Supplementary Information

Supplementary Note 1. Assessing relationships between covariates and social network position characteristics

Statistical tests were performed to investigate if covariates used in the current study were significantly associated with social network position characteristics. Associations between each social network position characteristic and continuous covariates were tested with Pearson correlations; associations between each social network position characteristic and categorical variables were assessed with *t*-tests.

Age. Age was not significantly associated with out-degree centrality (r = -0.017, p = 0.855), in-degree centrality (r = -0.106, p = 0.267), eigenvector centrality (r = -0.105, p = 0.271), betweenness centrality (r = 0.057, p = 0.551), or constraint (r = -0.089, p = 0.348).

Gender. Male and female subjects did not significantly differ in their out-degree centrality ($t(110)$ = 1.20, p = 0.232), in-degree centrality ($t(110)$ = 1.45, p = 0.783), eigenvector centrality ($t(110)$ = 1.45, p = 0.391), betweenness centrality ($t(110)$ = 1.45, p = 0.160), or constraint ($t(110)$ = 1.45, p = 0.151).

Handedness. Left-handed and right-handed subjects did not significantly differ in their out-degree centrality ($t(110)$ = 1.60, p = 0.874), in-degree centrality ($t(110)$ = -0.216 p = 0.830), eigenvector centrality ($t(110)$ = -0.226, p = 0.822), betweenness centrality ($t(110)$ = 0.765, p = 0.446), or constraint ($t(110)$ = -0.371, p = 0.711).

Cohort. Subjects in cohort 1 and subjects in cohort 2 did not significantly differ in their out-degree centrality ($t(110)$ = -0.848, p = 0.400), in-degree centrality ($t(110)$ = -0.994, p = 0.323), eigenvector centrality ($t(110)$ = -0.906, p = 0.368), betweenness centrality ($t(110)$ = -1.124, p = 0.265), or constraint ($t(110)$ = -0.526, p = 0.600). Subjects in cohort 1 and subjects in cohort 3 did not significantly differ in their out-degree centrality ($t(110)$ = -0.316, p = 0.753), in-degree centrality ($t(110)$ = -0.282, p = 0.779), eigenvector centrality ($t(110)$ = -0.055, p = 0.957), betweenness centrality ($t(110)$ = -0.754, p = 0.454), or constraint ($t(110)$ = -0.971, p = 0.335). Subjects in cohort 2 and subjects in cohort 3 did not significantly differ in their out-degree centrality ($t(110)$ = 0.507, p = 0.614), in-degree centrality ($t(110)$ = 0.691, p = 0.492), eigenvector centrality ($t(110)$ = 0.895, p = 0.373), betweenness centrality ($t(110)$ = 0.310, p = 0.757), or constraint ($t(110)$ = -0.604, p = 0.547). The lack of significant differences in social network position characteristics across cohorts is to be expected given, for example, that we normalized data within cohort prior to aggregating data across cohorts for subsequent analyses.

Extraversion. Extraversion was significantly associated with out-degree centrality (r = 0.264, p = 0.005), in-degree centrality (r = 0.481, $p = 8.00 \times 10^{-8}$), eigenvector centrality (r = 0.373, $p = 5.16 \times 10^{-5}$), betweenness centrality (r = 0.330, $p = 3.73 \times 10^{-4}$), and constraint (r = 0.316, $p = 6.95 \times 10^{-4}$).

Supplementary Note 2. Testing if patterns of white matter microstructure across major white matter tracts are predictive of social network position characteristics

We complemented our main analyses, which were specifically focused on tracts between regions implicated in social and affective processing, by conducting an exploratory analysis of major, well-established white matter tracts. To do so, we used Freesurfer's TRActs Constrained by UnderLying Anatomy (TRACULA) tool[1], an algorithm for automated global probabilistic tractography, which reconstructs 18 major white matter tracts for each subject. We then performed the following procedure to characterize the predictors used in the predictive modeling analysis. Each subject's FA map was thresholded at 0.20. For each subject, each tract was thresholded at 20% of the maximum value, binarized, and used as a mask to extract the mean FA from the corresponding subject's FA image. This procedure yielded 18 predictors that captured a pattern of white matter microstructural integrity distributed across major white matter tracts in each subject's brain.

Similar to our main analyses, we first implemented a data-driven, machine learning approach to predict individuals' social network position characteristics based on their patterns of microstructural integrity distributed across these 18 well-established white matter tracts using a ridge regression-based algorithm (see Methods in the main text). Using a leave-one-subject-out cross-validation scheme, the algorithm significantly predicted individuals' eigenvector centrality (r = 0.215, p = 0.011) and betweenness centrality (r = 0.186, p = 0.025) based on patterns of white matter microstructural integrity distributed across all 18 white matter tracts (see Methods). Each reported correlation value reflects the relationship between the actual social network position characteristic values and the values predicted by a given model. This analytical procedure was then repeated while controlling for demographic characteristics (age, gender), extraversion, handedness, and cohort. Patterns of microstructural integrity across the 18 major white matter tracts were significantly predictive of in-degree centrality (r = 0.233, p = 0.007) and eigenvector centrality (r = 0.291, p = 0.001) when controlling for these variables.

Assessing relations between social network position characteristics

Statistical tests were performed to investigate the relationships between the social network characteristics. Associations between each social network position were tested with Pearson correlations (Supplementary Table 1).

Supplementary Table 1. Correlations between social network characteristics.

	Out-degree centrality	In-degree centrality	Eigenvector centrality	Betweenness centrality
In-degree centrality	0.51***			
Eigenvector centrality	0.87***	0.80***		
Betweenness centrality	0.89***	0.59***	0.76***	
Constraint	0.73***	0.72***	0.81***	0.68***

Note: Constraint was negated to yield a measure of brokerage. ***$p < .001$

Supplementary Table 2. Functionally defined ROIs in the affective processing network.

ROI Label	Hemisphere	Voxel count	Region(s)	Notes
Aff-1	left	99	rostral anterior cingulate cortex	
Aff-2	left	273	ventromedial prefrontal cortex	
Aff-3	left	502	dorsomedial prefrontal cortex	
Aff-4	left	134	insula	
Aff-5	left	729	orbitofrontal cortex	
Aff-6	left	224	amygdala	Masked using anatomical amygdala mask
Aff-7	left	261	temporal pole	Masked using anatomical temporal pole mask
Aff-8	left	332	superior frontal gyrus	
Aff-9	left	209	dorsal-rostral anterior cingulate cortex	
Aff-10	right	243	amygdala	Masked using anatomical amygdala mask
Aff-11	right	86	caudal anterior cingulate cortex	
Aff-12	right	304	dorsomedial prefrontal cortex	
Aff-13	right	147	dorsal-rostral anterior cingulate cortex	
Aff-14	right	981	inferior frontal gyrus	
Aff-15	right	198	insula	
Aff-16	right	106	rostral anterior cingulate cortex	
Aff-17	right	498	temporal pole	Masked using anatomical temporal pole mask
Aff-18	right	262	ventromedial prefrontal cortex	

Note: Where noted, cortical ROIs that were additionally masked using anatomical masks based on the Desikan-Killiany atlas as implemented in FreeSurfer and subcortical ROIs that were additionally masked based on anatomical masks based on automatic subcortical segmentation in FreeSurfer (see Methods for more details)[2–4]. These masks were generated for each participant using FreeSurfer's recon-all command.

Supplementary Table 3. Functionally defined ROIs in the face processing network.

ROI Label	Hemisphere	Voxel count	Region(s)	Notes
Face-1	left	224	amygdala	Masked using anatomical amygdala mask
Face-2	left	803	inferior temporal gyrus	
Face-3	left	386	posterior superior temporal sulcus	
Face-4	left	459	occipital pole	
Face-5	left	90	lingual cortex	Masked using anatomical lingual cortex mask
Face-6	left	798	lateral occipital cortex	Masked using anatomical lateral occipital cortex mask
Face-7	left	892	fusiform cortex	Masked using anatomical fusiform cortex mask
Face-8	left	337	inferior temporal cortex	Masked using anatomical inferior temporal cortex mask
Face-9	right	243	amygdala	Masked using anatomical amygdala mask
Face-10	right	424	inferior frontal gyrus	
Face-11	right	104	lingual cortex	Masked using anatomical lingual cortex mask
Face-12	right	980	lateral occipital cortex	Masked using anatomical lateral occipital cortex mask
Face-13	right	808	fusiform cortex	Masked using anatomical fusiform cortex mask
Face-14	right	282	middle temporal cortex	Masked using anatomical middle temporal cortex mask
Face-15	right	484	inferior temporal cortex	Masked using anatomical inferior temporal cortex mask
Face-16	right	230	posterior superior temporal sulcus	Masked using anatomical superior temporal sulcus mask
Face-17	right	160	temporal pole	Masked using anatomical temporal pole mask
Face-18	right	354	anterior inferior temporal cortex	Masked using anatomical anterior inferior temporal cortex mask

Note: Where noted, cortical ROIs that were additionally masked using anatomical masks based on the Desikan-Killiany atlas as implemented in FreeSurfer and subcortical ROIs that were additionally masked based on anatomical masks based on automatic subcortical segmentation in FreeSurfer (see Methods for more details)[2–4]. These masks were generated for each participant using FreeSurfer's recon-all command.

Supplementary Table 4. Functionally defined ROIs in the mentalizing network.

ROI Label	Hemisphere	Voxel count	Region(s)	Notes
Ment-1	left	1013	precuneus	
Ment-2	left	586	dorsomedial prefrontal cortex	
Ment-3	left	341	ventromedial prefrontal cortex	
Ment-4	left	77	insula	
Ment-5	left	1661	angular gyrus	
Ment-6	left	733	supramaginal gyrus	
Ment-7	left	459	posterior superior temporal sulcus	
Ment-8	left	154	middle temporal gyrus	
Ment-9	left	676	temporal pole	
Ment-10	right	221	ventromedial prefrontal cortex	
Ment-11	right	820	dorsomedial prefrontal cortex	
Ment-12	right	1024	precuneus	
Ment-13	right	2127	inferior frontal gyrus, dorsal prefrontal cortex	
Ment-14	right	1169	temporal pole, anterior temporal cortex	
Ment-15	right	3374	occipitotemporal cortex	
Ment-16	right	1003	supramaginal gyrus	
Ment-17	right	282	posterior superior temporal sulcus	
Ment-18	right	388	supplementary motor cortex	

Note: Where noted, cortical ROIs that were additionally masked using anatomical masks based on the Desikan-Killiany atlas as implemented in FreeSurfer and subcortical ROIs that were additionally masked based on anatomical masks based on automatic subcortical segmentation in FreeSurfer (see Methods for more details)[2–4]. These masks were generated for each participant using FreeSurfer's recon-all command.

Supplementary Table 5. Functionally defined ROIs in the mirroring network.

ROI Label	Hemisphere	Voxel count	Region(s)	Notes
Mirr-1	left	1576	lateral occipital cortex	
Mirr-2	left	529	temporoparietal junction	
Mirr-3	left	1439	precentral gyrus, inferior frontal gyrus	
Mirr-4	left	2608	superior parietal lobule	
Mirr-5	left	956	supramarginal gyrus, postcentral gyrus	
Mirr-6	left	703	premotor cortex	
Mirr-7	right	291	supplementary motor cortex	
Mirr-8	right	347	precuneus	
Mirr-9	right	743	premotor cortex	
Mirr-10	right	1123	supramarginal gyrus	
Mirr-11	right	378	temporoparietal junction	
Mirr-12	right	390	lateral occipital cortex	
Mirr-13	right	330	lingual cortex	
Mirr-14	right	1053	precentral gyrus, inferior frontal gyrus	
Mirr-15	right	2766	occipitotemporal cortex	
Mirr-16	right	472	occipital pole	
Mirr-17	right	2872	superior parietal lobule	
Mirr-18	right	36	posterior cingulate cortex	
Mirr-19	right	542	supramarginal gyrus, postcentral gyrus	

Note: Where noted, cortical ROIs that were additionally masked using anatomical masks based on the Desikan-Killiany atlas as implemented in FreeSurfer and subcortical ROIs that were additionally masked based on anatomical masks based on automatic subcortical segmentation in FreeSurfer (see Methods for more details)[2–4]. These masks were generated for each participant using FreeSurfer's recon-all command.

Supplementary References

1. Yendiki, A. *et al.* Automated probabilistic reconstruction of white-matter pathways in health and disease using an atlas of the underlying anatomy. *Front. Neuroinform.* **5**, 5–23 (2011).
2. Fischl, B. *et al.* Whole brain segmentation: automated labeling of neuroanatomical structures in the human brain. *Neuron* **33**, 341–355 (2002).
3. Fischl, B. FreeSurfer. *Neuroimage* **62**, 774–781 (2012).
4. Desikan, R. S. *et al.* An automated labeling system for subdividing the human cerebral cortex on MRI scans into gyral based regions of interest. *Neuroimage* **31**, 968–980 (2006).
