# Supplementary material for: White matter connectivity in brain networks supporting social and affective processing predicts real-world social network characteristics
Source: Commun Biol. 2022 Oct 3;5:1048. doi: 10.1038/s42003-022-03655-8 (PMC9529948; doi:10.1038/s42003-022-03655-8)
Supplement: Supplementary file 3 — Description of Additional Supplementary Files [file 42003_2022_3655_MOESM3_ESM.pdf]

Description of Additional Supplementary Files

File name: Supplementary Data 1
Description: Data for each of the scatterplots and histograms have been provided in an Excel file. For each scatterplot, the predicted and actual values have been provided. For each histogram, the permuted values have been provided. For each plot, there is a worksheet labeled by the brain network, social network position characteristic, and plot type (i.e., scatterplot or histogram).
