# Supplementary material for: White matter connectivity in brain networks supporting social and affective processing predicts real-world social network characteristics
Source: Commun Biol. 2022 Oct 3;5:1048. doi: 10.1038/s42003-022-03655-8 (PMC9529948; doi:10.1038/s42003-022-03655-8)
Supplement: Supplementary file 5 — Reporting Summary [file 42003_2022_3655_MOESM5_ESM.pdf]

nature portfolio

Corresponding author(s): Ryan Hyon and Carolyn Parkinson

Last updated by author(s): Jul 12, 2021

Reporting Summary

Nature Portfolio wishes to improve the reproducibility of the work that we publish. This form provides structure for consistency and transparency in reporting. For further information on Nature Portfolio policies, see our Editorial Policies and the Editorial Policy Checklist.

Statistics

For all statistical analyses, confirm that the following items are present in the figure legend, table legend, main text, or Methods section.

n/a	Confirmed	
☐	☒	The exact sample size (*n*) for each experimental group/condition, given as a discrete number and unit of measurement
☐	☒	A statement on whether measurements were taken from distinct samples or whether the same sample was measured repeatedly
☐	☒	The statistical test(s) used AND whether they are one- or two-sided<br>*Only common tests should be described solely by name; describe more complex techniques in the Methods section.*
☐	☒	A description of all covariates tested
☐	☒	A description of any assumptions or corrections, such as tests of normality and adjustment for multiple comparisons
☐	☒	A full description of the statistical parameters including central tendency (e.g. means) or other basic estimates (e.g. regression coefficient) AND variation (e.g. standard deviation) or associated estimates of uncertainty (e.g. confidence intervals)
☐	☒	For null hypothesis testing, the test statistic (e.g. *F*, *t*, *r*) with confidence intervals, effect sizes, degrees of freedom and *P* value noted<br>*Give P values as exact values whenever suitable.*
☒	☐	For Bayesian analysis, information on the choice of priors and Markov chain Monte Carlo settings
☒	☐	For hierarchical and complex designs, identification of the appropriate level for tests and full reporting of outcomes
☐	☒	Estimates of effect sizes (e.g. Cohen's *d*, Pearson's *r*), indicating how they were calculated

Our web collection on statistics for biologists contains articles on many of the points above.

Software and code

Policy information about availability of computer code

Data collection	MRI data were acquired using a Philips Achieva 3.0 Tesla scanner using a 32-channel phased array head coil. Philips software was used at the scanner console during data collection.
Data analysis	Data were analyzed using open-source software in R and Python. Packages used include igraph (for social network analysis), scikit-learn (for predictive modeling), and seaborn (for data visualization). Preprocessing, ROI definition, and probabilistic tractography were performed using FSL. Neurosynth was also used as the basis for ROI definition.

For manuscripts utilizing custom algorithms or software that are central to the research but not yet described in published literature, software must be made available to editors and reviewers. We strongly encourage code deposition in a community repository (e.g. GitHub). See the Nature Portfolio guidelines for submitting code & software for further information.

Data

Policy information about availability of data

All manuscripts must include a data availability statement. This statement should provide the following information, where applicable:

- Accession codes, unique identifiers, or web links for publicly available datasets
- A description of any restrictions on data availability
- For clinical datasets or third party data, please ensure that the statement adheres to our policy

The data and code that support the findings of this study are available upon request.

Field-specific reporting

Please select the one below that is the best fit for your research. If you are not sure, read the appropriate sections before making your selection.

☐ Life sciences ☒ Behavioural & social sciences ☐ Ecological, evolutionary & environmental sciences

For a reference copy of the document with all sections, see nature.com/documents/nr-reporting-summary-flat.pdf

Behavioural & social sciences study design

All studies must disclose on these points even when the disclosure is negative.

Study description	This is a quantitative study involving tests for association between social network position characteristics and patterns of white matter microstructural integrity.
Research sample	Subjects in the social network survey portion of the study were from three different cohorts of first-year students in a graduate program at a private university in the United States who participated as part of their coursework on leadership. The total size of all three cohorts was 842 students, and 839 students participated in the social network survey, resulting in an overall response rate of 99.6% (N[Cohort-1] = 275, 91 females, response rate = 99.3%; N[Cohort-2] = 279, 89 females, response rate = 100%; N[Cohort-3] = 285, 120 females, response rate = 99.7%). A subset of 130 individuals who had completed the social network survey completed a subsequent dMRI study (n[Cohort-1] = 54; n[Cohort-2] = 36; n[Cohort-3] = 40). Of the 130 neuroimaging subjects, data from 18 subjects were excluded from analysis due to excess movement. Data from the resulting 112 subjects (40 female) aged 24-35 (M = 27.78, SD = 2.01) were used for subsequent neuroimaging analyses.
Sampling strategy	We sought to recruit as many participants as possible who were members of three different first-year cohorts of students in a graduate program in a rural, isolated location who largely live, eat, socialize, and study with one another (Dartmouth College's Tuck School of Business). Students were selected from these cohorts because the full social networks of each of these bounded communities could be characterized and because these communities tend to constitute most or all of participants' "social worlds" during their time in the program. All students in these cohorts who were interested in participating in the study were eligible to do so, as long as they did not have any contraindications for MRI scanning (e.g., related to scanner safety). Our final neuroimaging sample size of 130 (112 after exclusions) is consistent with the approximate sample sizes of other recent studies linking white matter connectivity and individual difference variables.
Data collection	The social-network survey was conducted online via participants' computers, and no researchers were present during data collection. Neuroimaging data were collected using a Philips Achieva 3.0 Tesla scanner using a 32-channel phased array head coil and two researchers were present for all data analysis (due to the safety protocol of the scanning center). While the researchers were aware of the general hypotheses that were being tested in the study, the researchers were not aware of participants' social-network centralities during data collection.
Timing	Cohort 1's data was collected between April and May 2014. Cohort 2's data was collected between February and March 2015. Cohort 3's data was collected in August 2015.
Data exclusions	Of the 130 subjects in Part 2, data from 18 subjects were excluded from analysis due to excess movement.
Non-participation	No participants dropped out of the study.
Randomization	Not applicable as the study is purely correlational.

Reporting for specific materials, systems and methods

We require information from authors about some types of materials, experimental systems and methods used in many studies. Here, indicate whether each material, system or method listed is relevant to your study. If you are not sure if a list item applies to your research, read the appropriate section before selecting a response.

Materials & experimental systems

n/a	Involved in the study	
☐	☐	Antibodies
☐	☐	Eukaryotic cell lines
☐	☐	Palaeontology and archaeology
☐	☐	Animals and other organisms
☐	☒	Human research participants
☐	☐	Clinical data
☐	☐	Dual use research of concern

Methods

n/a	Involved in the study	
☒	☐	ChIP-seq
☒	☐	Flow cytometry
☐	☒	MRI-based neuroimaging

Antibodies

Antibodies used	*Describe all antibodies used in the study; as applicable, provide supplier name, catalog number, clone name, and lot number.*
Validation	*Describe the validation of each primary antibody for the species and application, noting any validation statements on the manufacturer's website, relevant citations, antibody profiles in online databases, or data provided in the manuscript.*

Eukaryotic cell lines

Policy information about cell lines

Cell line source(s)	*State the source of each cell line used.*
Authentication	*Describe the authentication procedures for each cell line used OR declare that none of the cell lines used were authenticated.*
Mycoplasma contamination	*Confirm that all cell lines tested negative for mycoplasma contamination OR describe the results of the testing for mycoplasma contamination OR declare that the cell lines were not tested for mycoplasma contamination.*
Commonly misidentified lines (See ICLAC register)	*Name any commonly misidentified cell lines used in the study and provide a rationale for their use.*

Palaeontology and Archaeology

Specimen provenance	*Provide provenance information for specimens and describe permits that were obtained for the work (including the name of the issuing authority, the date of issue, and any identifying information). Permits should encompass collection and, where applicable, export.*
Specimen deposition	*Indicate where the specimens have been deposited to permit free access by other researchers.*
Dating methods	*If new dates are provided, describe how they were obtained (e.g. collection, storage, sample pretreatment and measurement), where they were obtained (i.e. lab name), the calibration program and the protocol for quality assurance OR state that no new dates are provided.*

☐ Tick this box to confirm that the raw and calibrated dates are available in the paper or in Supplementary Information.

Ethics oversight	*Identify the organization(s) that approved or provided guidance on the study protocol, OR state that no ethical approval or guidance was required and explain why not.*

Note that full information on the approval of the study protocol must also be provided in the manuscript.

Animals and other organisms

Policy information about studies involving animals; ARRIVE guidelines recommended for reporting animal research

Laboratory animals	*For laboratory animals, report species, strain, sex and age OR state that the study did not involve laboratory animals.*
Wild animals	*Provide details on animals observed in or captured in the field; report species, sex and age where possible. Describe how animals were caught and transported and what happened to captive animals after the study (if killed, explain why and describe method; if released, say where and when) OR state that the study did not involve wild animals.*
Field-collected samples	*For laboratory work with field-collected samples, describe all relevant parameters such as housing, maintenance, temperature, photoperiod and end-of-experiment protocol OR state that the study did not involve samples collected from the field.*
Ethics oversight	*Identify the organization(s) that approved or provided guidance on the study protocol, OR state that no ethical approval or guidance was required and explain why not.*

Note that full information on the approval of the study protocol must also be provided in the manuscript.

Human research participants

Policy information about studies involving human research participants

Population characteristics	See above.
Recruitment	Participants who were students in three large graduate student cohorts in a rural, isolated location who largely live, eat, socialize, and study with one another (Dartmouth College's Tuck School of Business). Students were selected from these cohorts because the full social networks of each of these bounded communities had been characterized. All students in these cohorts who were interested in participating in the study were eligible to do so, as long as they did not have any contraindications for MRI scanning (e.g., related to scanner safety).
Ethics oversight	Institutional Review Board of Dartmouth College

Note that full information on the approval of the study protocol must also be provided in the manuscript.

Clinical data

Policy information about clinical studies

All manuscripts should comply with the ICMJE guidelines for publication of clinical research and a completed CONSORT checklist must be included with all submissions.

Clinical trial registration	*Provide the trial registration number from ClinicalTrials.gov or an equivalent agency.*
Study protocol	*Note where the full trial protocol can be accessed OR if not available, explain why.*
Data collection	*Describe the settings and locales of data collection, noting the time periods of recruitment and data collection.*
Outcomes	*Describe how you pre-defined primary and secondary outcome measures and how you assessed these measures.*

Dual use research of concern

Policy information about dual use research of concern

Hazards

Could the accidental, deliberate or reckless misuse of agents or technologies generated in the work, or the application of information presented in the manuscript, pose a threat to:

No	Yes	
☒	☐	Public health
☒	☐	National security
☒	☐	Crops and/or livestock
☒	☐	Ecosystems
☒	☐	Any other significant area

Experiments of concern

Does the work involve any of these experiments of concern:

No	Yes	
☒	☐	Demonstrate how to render a vaccine ineffective
☒	☐	Confer resistance to therapeutically useful antibiotics or antiviral agents
☒	☐	Enhance the virulence of a pathogen or render a nonpathogen virulent
☒	☐	Increase transmissibility of a pathogen
☒	☐	Alter the host range of a pathogen
☒	☐	Enable evasion of diagnostic/detection modalities
☒	☐	Enable the weaponization of a biological agent or toxin
☒	☐	Any other potentially harmful combination of experiments and agents

Magnetic resonance imaging

Experimental design

Design type	Structural neuroimaging study (no task data used)
Design specifications	No blocks or trials used - Structural neuroimaging study.
Behavioral performance measures	No behavioral performance measures used (structural neuroimaging study).

Acquisition

Imaging type(s)	Diffusion
Field strength	3T
Sequence & imaging parameters	Diffusion-weighted images were collected using 70 contiguous 2 mm thick axial slices with 32 diffusion directions (91 ms TE, 8845 TR, 1000 s/mm2 b-value, 240 mm FOV, 90° flip angle, 1.875 mm x 1.875 mm x 2 mm voxel size). High-resolution anatomical images were also acquired using a T1-weighted MPRAGE protocol (8.2 s TR; 3.7 ms TE; 240 x 187

FOV; 0.938 mm x 0.938 mm x 1.0 mm).

Area of acquisition: A whole brain scan was used.

Diffusion MRI ☒ Used ☐ Not used

Parameters: See "Sequence & imaging parameters" above.

Preprocessing

Preprocessing software: Data were preprocessed using FSL 5.0.10. Diffusion-weighted images were corrected for eddy currents and head motion using FSL eddy. Brain extraction was performed using FSL BET.

Normalization: Both linear and non-linear methods were used to align subjects' fractional anisotropy images in native space to MNI152 standard space. FSL FLIRT (12 degrees of freedom, corratio cost function) was used to generate an affine transformation matrix to align fractional anisotropy images to T1 anatomical images. FSL FLIRT was used to generate an affine transformation matrix to align T1 space to MNI152 standard space, and FSL FNIRT used this "affine guess" to generate a non-linear warpfield to align T1 space to MNI152 standard space. FSL FNIRT was then used to apply the first affine FLIRT matrix (i.e., native space to T1 space transform) and the FNIRT warpfield in one step to transform fractional anisotropy images to MNI152 standard space.

Normalization template: Data were normalized to MNI152 space.

Noise and artifact removal: FSL eddy was used to correct eddy currents and subject movement in the diffusion-weighted data. Subjects that exhibited more than 2mm of movement between volumes were excluded from analysis.

Volume censoring: No volume censoring was performed.

Statistical modeling & inference

Model type and settings: Multivariate analyses were used, as described in the "Multivariate modeling and predictive analysis" field below and in the "Structural connectome-based predictive modeling of social network position characteristics" sub-section of the Methods section of the manuscript.

Effect(s) tested: Using ridge regression, we tested if patterns of white matter microstructure, distributed across tracts within social and affective processing systems, are predictive of social network position characteristics.

Specify type of analysis: ☐ Whole brain ☒ ROI-based ☐ Both

Anatomical location(s): As described in the "ROI definition" sub-section of the Methods section, we first defined cortical ROIs using Neurosynth. We then performed probabilistic tractography to reconstruct tracts between pairs of ROIs; FA values in these tracts were the basis of our primary analyses.

Statistic type for inference (See Eklund et al. 2016): We note that our analyses, in which we tested if distributed patterns of white matter microstructural integrity are predictive of trait variables, are not impacted by the concerns that the Eklund et al. (2016) paper raised regarding inflated false-positive rates in fMRI inferences for spatial extent. Correction for multiple comparisons across statistical tests was implemented using False-Discovery Rate (FDR) correction (as specified in the "Correction" field below).

Correction: We used FDR correction for all analyses.

Models & analysis

n/a	Involved in the study
☒	☐ Functional and/or effective connectivity
☐	☒ Graph analysis
☐	☒ Multivariate modeling or predictive analysis

Graph analysis: No graph analysis was done on the neuroimaging data but the unweighted graph of each cohort's social network was characterized. Node-level characteristics used were: out-degree centrality, in-degree centrality, eigenvector centrality, betweenness centrality, and constraint.

Multivariate modeling and predictive analysis: Independent variables: FA values in each tract in the set of tracts used in each analysis. Model: Ridge regression using nested cross-validation to perform hyperparameter tuning. Training and evaluation metrics: correlation between predicted and actual social network position characteristics values. See "Structural connectome-based predictive modeling of social network position characteristics" sub-section of the Methods section for more details.
